# Distributional characteristics analysis of aeroallergens among children With allergic respiratory diseases in Sichuan province, China

**DOI:** 10.3389/fped.2026.1829893

**Published:** 2026-05-18

**Authors:** Ling Liao, Lamei Liu, Qi Wang, Shujing Gao

**Affiliations:** 1 Department of Pediatric Respiratory Nursing, West China Second University Hospital, Sichuan University, Chengdu, Sichuan, China; 2 Key Laboratory of Birth Defects and Related Diseases of Women and Children (Sichuan University), Ministry of Education, Chengdu, Sichuan, China

**Keywords:** aeroallergens, allergic respiratory disease, children, cross-sectional study, skin prick test

## Abstract

**Objective:**

To analyze the prevalence and distribution patterns of aeroallergens in children with respiratory allergic diseases in Sichuan Province based on skin prick test (SPT) results.

**Methods:**

This retrospective cross-sectional study included 7,301 pediatric patients with respiratory allergic diseases who attended the outpatient department of West China Second University Hospital between January 2023 and December 2024. All patients underwent standardized skin prick testing.

**Results:**

Overall, 52.4% of children showed positive SPT results. The three most prevalent sensitizing allergens were *Dermatophagoides pteronyssinus (Der.p*), *Dermatophagoides farinae* (*Der.f*), and house dust. Among the 3,826 children with positive SPT results, 86.5% were sensitized to two or more allergens. Most allergens exhibited the highest positive rates in the Sichuan Basin. With increasing age, sensitization rates to most inhalant allergens showed an upward trend. Across all age groups and disease categories, *Der.f*, *Der.p*, and house dust remained the most frequently detected allergens. Preschool children had the lowest overall SPT positivity rates. In addition, children with allergic rhinitis and those with concomitant atopic diseases showed significantly higher sensitization rates and broader polysensitization profiles than children with asthma alone or without such comorbidities.

**Conclusion:**

*Der.p* and *Der.f* are the predominant airborne allergens among children with respiratory allergic diseases in Sichuan Province, likely influenced by the region's humid basin climate and geographic heterogeneity. Sensitization patterns vary significantly according to age, disease phenotype, geographic region, season, and the presence of concomitant atopic diseases. These findings provide important region-specific evidence for optimized allergen screening, early risk stratification, and individualized immunotherapy strategies in pediatric clinical practice.

## Key message

This large-scale retrospective study of 7,301 children with respiratory allergic diseases in Sichuan Province revealed that *Dermatophagoides pteronyssinus* and *Dermatophagoides farinae* were the predominant aeroallergens, followed by house dust. The majority of sensitized children exhibited multiple allergen sensitization, and the overall positive rates increased with age. Importantly, children with other atopic diseases showed higher sensitization rates to major aeroallergens, suggesting that comorbid atopic conditions may be associated with enhanced allergic sensitization and represent a higher-risk subgroup for progressive allergic disease. These findings emphasize the regional dominance of mite sensitization and the influence of local climate, geography, and patient-related factors on pediatric allergic disease patterns, while also providing region-specific evidence for optimized allergen screening and individualized immunotherapy strategies.

## 1 Introduction

Allergic diseases encompass a group of immune-mediated hypersensitivity disorders elicited upon exposure to specific allergens via contact, ingestion, or inhalation (1). These conditions can affect multiple organ systems, with the respiratory tract, skin, and gastrointestinal tract being the most frequently involved. In addition to substantially compromising quality of life, allergic diseases impose significant economic burdens on both families and healthcare systems and, in severe instances, can be life-threatening (2). Due to their chronic nature and high recurrence rate, they pose a substantial and escalating challenge to global public health.

Respiratory allergies represent the most common form of allergic disorders, with allergic rhinitis (AR) and asthma (AS) being the two predominant manifestations, especially in pediatric populations. Epidemiological studies indicate that the global prevalence of AR in children is approaching 40% (3). In China, the prevalence of AR among children reached 18.46% in 2021 and continues to exhibit a steady increase (4). According to the Global Burden of Disease (GBD) database, around 97.5 million children worldwide were affected by asthma in 2021, including 9.4 million cases in China (5). The prevalence of asthma among children in major Chinese urban areas has also risen significantly, with cumulative incidence increasing from 1.09% to 3.02% over the past three decades. Repeated nationwide surveys consistently corroborate this upward trend in childhood asthma (6–8). Furthermore, AR and AS are closely interrelated: AR is widely recognized as a significant risk factor for the development of AS, and epidemiological data reveal substantial comorbidity between the two conditions. It is estimated that approximately 30% of patients with rhinitis eventually progress to asthma, while up to 80% of those with persistent asthma also present with rhinitis (9).

Allergens, also known as antigens, act as both pathogenic factors and triggers in respiratory allergic diseases, and sensitization to inhaled allergens is a key determinant of the development of airway disorders (10, 11). Upon re-exposure to sensitizing allergens, inflammatory mediators such as histamine are released, initiating a cascade of allergic responses that include bronchospasm and mucosal edema (12). Extensive research has demonstrated that sensitization profiles exhibit considerable geographic, climatic, and seasonal variation, reflecting the extensive diversity of inhalant allergens (13–15).

China encompasses a vast geographical area with extensive latitudinal coverage, complex topography, and marked climatic variations across regions. Although several nationwide surveys have reported allergen sensitization patterns in China, region-specific data focusing on pediatric respiratory allergic diseases remain limited. However, updated data on sensitization patterns to inhalant allergens among the pediatric population in Sichuan Province remain scarce. In particular, the complex climatic and topographical characteristics of Sichuan Province may lead to distinct aeroallergen exposure patterns that differ from those reported in other regions. The skin prick test (SPT), a standard diagnostic procedure for detecting specific IgE-mediated sensitization, provides a rapid and reliable method for allergen screening (16). It is especially useful in patients with allergic rhinitis and asthma for identifying causative allergens, thus supporting the development of personalized avoidance strategies and guiding specific immunotherapy.

Therefore, this study aims to investigate the prevalence and sensitization profiles of inhalant allergens among children in Sichuan province by employing a large-scale SPT. It further seeks to analyze variations in sensitization across demographic characteristics, allergic disease subtypes, and geographical and climatic factors, thereby providing a scientific basis for the early prevention and management of pediatric respiratory allergic diseases in the region.

## 2 Methods

### 2.1 Study participants

This retrospective cross-sectional study enrolled pediatric patients with respiratory conditions who attended the outpatient department of West China Second University Hospital between January 2023 and December 2024.

The inclusion criteria were as follows: (1) diagnosis of allergic rhinitis (AR) or asthma, established according to the Chinese Guideline for Diagnosis and Treatment of Allergic Rhinitis and the Guideline for the Diagnosis and Optimal Management of Asthma in Children (2016), respectively; (2) age between 0 and 18 years, with skin prick testing performed; and (3) availability of complete medical records, including age, sex, home address, diagnosis, clinical history, and SPT results.

Patients were excluded if they met any of the following criteria: (1) severe dysfunction of major organs (e.g., heart, liver, or kidneys); (2) autoimmune diseases or immunodeficiency disorders; (3) immune dysfunction resulting from prolonged use of corticosteroids or immunosuppressants; or (4) discontinuation of oral antihistamines or topical corticosteroids for less than one week prior to testing.

Children diagnosed with isolated non-respiratory allergic conditions (e.g., food allergy or atopic dermatitis without concomitant AR or asthma) were excluded from the main analyses.

### 2.2 Data collection

Eligible cases were identified from the electronic medical record system. Relevant patient information was extracted, and a structured database was constructed following a process of dual-entry verification, collation, and integration. All data were recoded to ensure anonymity, completeness, and accuracy.

Patients were categorized into three age groups according to commonly accepted pediatric developmental stages, as described in *Nelson Textbook of Pediatrics* and supported by previous epidemiological studies on pediatric allergic diseases (17, 18). The age groups were defined as early childhood (0–6 years), school-aged children (7–12 years), and adolescents (13–18 years). Although four children were recorded as 0 years of age, all were older than 28 days and therefore were not neonatal cases. They were also classified by clinical diagnosis into the following groups: allergic rhinitis (AR), asthma (AS), and comorbid asthma with allergic rhinitis (AS + AR).

### 2.3 Geographic and climatic classification

According to the residential addresses recorded in the electronic medical records, participants were classified into three geographic-climatic regions based on the topographic and climatic characteristics of Sichuan Province: the Sichuan Basin, the mountainous regions of southern Sichuan, and the plateau areas of northern Sichuan. This classification was based on the regional geographic division commonly used in regional epidemiological and climatological studies in Sichuan Province.

### 2.4 Skin prick test

Skin prick testing was performed by a trained operator under the supervision of a respiratory physician, following a standardized protocol. The volar forearm was cleansed with 75% ethanol or normal saline and allowed to air-dry. Subsequently, a positive control (histamine), a negative control (saline), and a panel of allergen extracts were applied. A sterile lancet was held at a 90-degree angle to the skin and used to prick through each droplet with a quick, firm motion, maintaining contact for approximately one second to ensure epidermal penetration. A new, single-use lancet was employed for each allergen to prevent cross-contamination. Patients or their guardians were instructed to keep the forearm extended with the palm facing upward during a 15–20 min observation period. The skin index (SI) was calculated as the mean wheal diameter of the allergen divided by the mean wheal diameter of the histamine positive control. Sensitization levels were graded as follows: SI = 0 (negative); Level 1 (0 < SI < 0.5, “+”); Level 2 (0.5 ≤ SI < 1.0, “++”); Level 3 (1.0 ≤ SI < 2.0, “+++”); Level 4 (SI ≥ 2.0, “++++”). Reactions graded from “+” to “++++” were considered positive, and grades “+++” or “++++” were defined as strongly positive. The allergen panel comprised ten common inhalant allergens: *Dermatophagoides farinae* (*Der.f*), *Dermatophagoides pteronyssinus* (*Der.p*), Cat dander, Dog hair, Cockroach, *Penicillium*, *Japanese hop*, House dust, *Artemisia*, and Feather. Multiple sensitization was defined as a positive skin reaction to two or more distinct allergens.

### 2.5 Statistics

Statistical analyses were conducted using SPSS Statistics(version 27.0; IBM Corp). Categorical variables were expressed as numbers and percentages. Group comparisons for categorical data were performed using the Chi-square test, with Fisher's exact test applied when expected cell frequencies were below 5. The Bonferroni method was employed to adjust for multiple comparisons. Multivariable binary logistic regression analysis was conducted to identify factors associated with positive skin prick test (SPT) results, with odds ratios (ORs) and 95% confidence intervals (CIs) reported. Additional multivariable binary logistic regression analyses were performed to assess whether concomitant atopic diseases were independently associated with sensitization to major aeroallergens after adjustment for age and sex. A two-sided *p*-value of < 0.05 was defined as the threshold for statistical significance.

### 2.6 Ethical considerations

This study was approved by the Ethics Committee of West China Second University Hospital, Sichuan University (Approval No. 2025-IRB-249). The requirement for written informed consent was waived by the Ethics Committee due to the retrospective study design and the use of fully anonymized patient data. No additional ethics approval was required in accordance with local and national guidelines.

### 2.7 Data availability statement

The data that support the findings of this study are not publicly available due to privacy and ethical restrictions related to patient information. Anonymized datasets are available from the corresponding author upon reasonable request.

## 3 Results

### 3.1 Participant characteristics

Between January 2023 and December 2024, a total of 7,301 patients (6.5 ± 2.9 years) were enrolled in this study. The cohort comprised 4,572 boys (62.6%, 6.6 ± 2.9 years) and 2,729 girls (37.4%, 6.3 ± 2.8 years). Based on clinical diagnosis, 5,475 patients (75.0%) were identified with allergic rhinitis (AR), 1,103 (15.1%) with asthma (AS), 723 (9.9%) with comorbid asthma and AR (AS and AR) (Table 1).

**Table 1:** Characteristics of participants.

Variable	Number (*n* = 7,301)		SPT-positive subjects			
	n	%	n	%	*X* ^2^	*P*
Overall SPT-positive cases			3,826	52.4		
Gender					19.045	<0.01^#^
Male	4,572	62.6	2,486	54.4		
Female	2,729	37.4	1,340	49.1		
Age(years)					139.750	<0.01^#^
0–6	4,138	56.7	1,919	46.4		
7–12	2,930	40.1	1,761	60.1		
13–18	233	3.2	146	62.7		
Disease					1,404.752	<0.01^#^
Allergic rhinitis	5,475	75.0	3,523	64.3		
Asthma	1,103	15.1	54	4.9		
Asthma with allergic rhinitis	723	9.9	249	34.4		
Region					301.807	<0.01^#^
Sichuan Basin	6,795	93.1	3,749	55.2		
Mountainous regions of southern Sichuan	337	4.6	48	14.2		
Plateau areas of northern Sichuan	169	2.3	29	17.2		
Season					26.834	<0.01^#^
Spring	1,581	21.7	777	49.1		
Summer	2,651	36.3	1,469	55.4		
Autumn	1,907	26.1	1,023	53.6		
Winter	1,162	15.9	557	47.9		

# *P* < 0.01.

**Figure 1:**
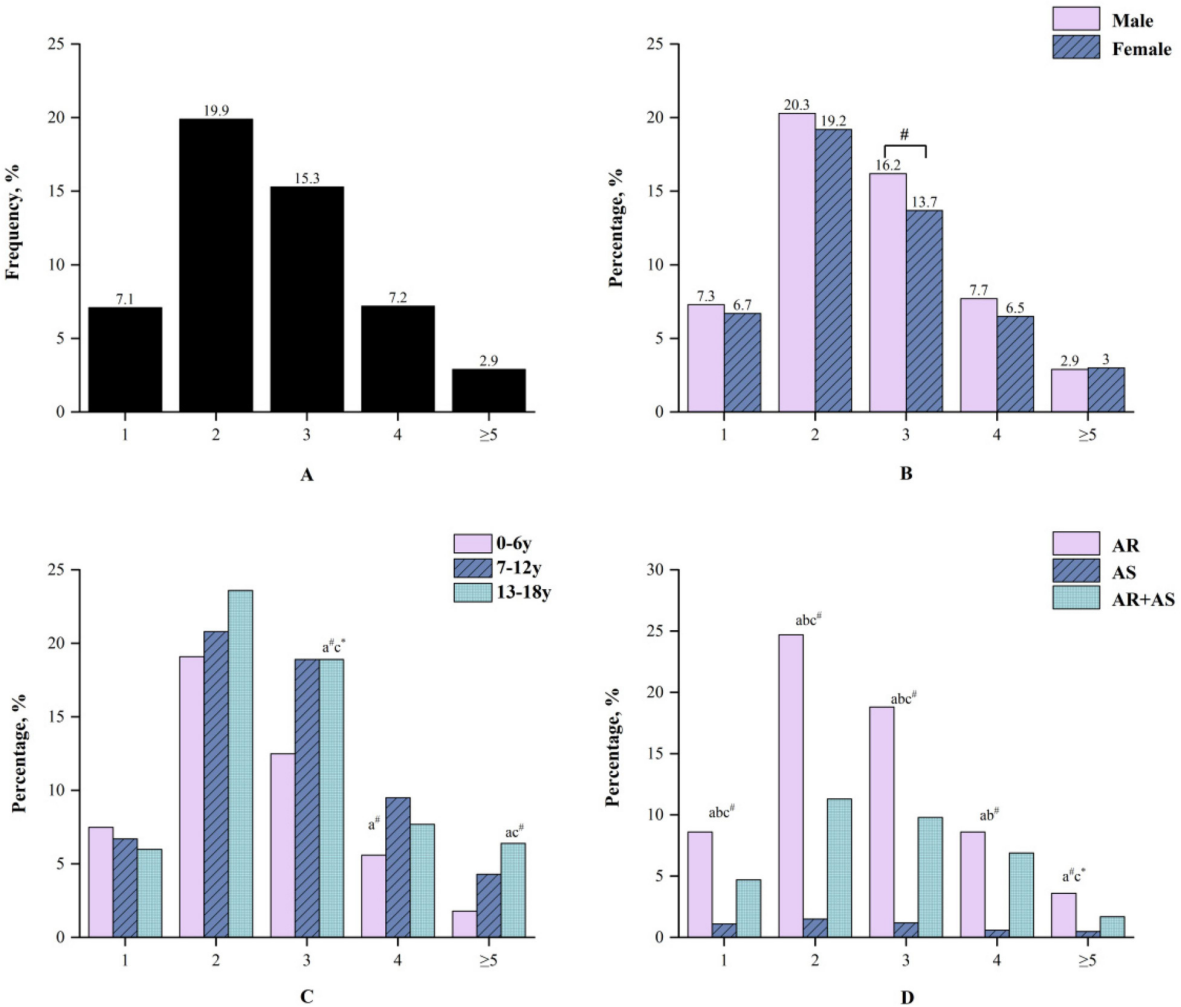
Sensitization to different number of positive allergens in different groups. **(A)** The number of people in different number of positive allergens in all person, **(B)** The number of positive allergens in different genders. **(C)** The number of positive allergens in different ages. **(a)** Compared between 0 and 6 years group and 7–12 years group. **(b)** Compared between 7 and 12 years group and 13–18 years group. **(c)** Compared between 0 and 6 years group and 13–18 years group. **(D)** The number of positive allergens in different diseases. **(a)** Compared between AR group and asthma group. **(b)** Compared between asthma group and AR combined with asthma group. c: Compared between AR group and AR combined with asthma group. *P < 0.05. ^#^*P* < 0.01.

Of the 7,301 participants, 3,826 (52.4%) showed a positive skin reaction to at least one allergen, as summarized in Table 1. The top three allergens with the highest proportions in both SPT sensitization rates and strong positive reactions were *Der.p*, *Der.f*, and house dust (Table 2). 518 (7.1%) cases were sensitized to one allergen, 3,308 (45.3%) cases were sensitized to 2 or more allergens (Figure 1A).

**Table 2:** Positive rates and distribution of various allergens in skin prick test in patients.

Allergen Types	Skin Prick Test					Positive Number		Strong Positve Number	
	**−**	**+**	**++**	**+++**	**++++**	N	%	N	%
*Der.p*	3,945	247	1,034	1,591	484	3,356	46.0	2,075	28.4
*Der.f*	3,990	363	1,085	1,476	387	3,311	45.3	1,863	25.5
House dust	6,048	404	648	189	12	1,253	17.2	201	2.8
Cat dander	6,813	162	238	80	8	488	6.7	88	1.2
Dog hair	6,834	154	217	91	5	467	6.4	96	1.3
Cockroach	6,881	159	214	44	3	420	5.8	47	0.6
*Japanese hop*	6,907	101	137	100	56	394	5.4	156	2.1
*Artemisia*	7,097	62	78	51	13	204	2.8	64	0.9
*Penicillium*	7,223	35	31	10	2	78	1.1	12	0.2
Feather	7,254	16	26	5	0	47	0.6	5	0.1

*Der.p, Dermatophagoides pteronyssinus, Der.f, Dermatophagoides farinae.*

### 3.2 Differences in allergic sensitizations among different groups

**Gender differences:** The overall SPT positivity rate was significantly higher in boys (54.4%) than in girls (49.1%, *P* < 0.01) (Table 1). Boys also showed significantly higher sensitization rates to *Der.p*, *Der.f*, house dust, and *Artemisia*. In addition, male patients had a higher frequency of strongly positive reactions (grade “+++” or “++++”) to *Der.p* and *Der.f* compared to females. No significant gender differences were observed for the other six inhalant allergens (Table 3). Furthermore, boys exhibited a significantly higher proportion of polysensitization to three or more allergens than girls (*P* < 0.01) (Figure 1B).

**Table 3:** Comparison of sensitized allergens between different groups.

Parameters	*Der.f*	*Der.p*	House dust	Cat dander	Dog hair	Cockroach	*Japanese hop*	*Artemisia*	*Penicillium*	Feather
Gender										
Male	2,136	2,182	823	326	298	272	263	142	53	26
%	46.7	47.7	18	7.1	6.5	5.9	5.8	3.1	1.2	0.6
Female	1,175	1,174	430	162	169	148	131	62	25	21
%	43.1	43	15.8	5.9	6.2	5.4	4.8	2.3	0.9	0.8
* P*	0.002^#^	<0.01^#^	0.015*	0.053	0.621	0.377	0.087	0.04*	0.349	0.295
Diseases										
AR	3,074	3,089	1,156	439	415	388	348	180	65	42
%	56.1	56.4	21.1	8.0	7.6	7.1	6.4	3.3	1.2	0.8
AS	38	48	16	12	12	6	4	3	5	2
%	3.4	4.4	1.5	1.1	1.1	0.5	0.4	0.3	0.5	0.2
AR + AS	199	219	81	37	40	26	42	21	8	3
%	27.5	30.3	11.2	5.1	5.5	3.6	5.8	2.9	1.1	0.4
*P*	<0.01^#^	<0.01^#^	<0.01^#^	<0.01^#^	<0.01^#^	<0.01^#^	<0.01^#^	<0.01^#^	0.096	0.061
Geographic regions										
Basin	3,259	3,304	1,236	475	459	412	374	185	76	46
%	48	48.6	18.2	7	6.8	6.1	5.5	2.7	1.1	0.7
Mountain	35	36	14	6	5	6	8	8	2	0
%	10.4	10.7	4.2	1.8	1.5	1.8	2.4	2.4	0.6	0
Plateau	17	16	3	7	3	2	12	11	0	1
%	10.1	9.5	1.8	4.1	1.8	1.2	7.1	6.5	0	0.6
* P*	<0.01^#^	<0.01^#^	<0.01^#^	<0.01^#^	<0.01^#^	<0.01^#^	0.028*	0.012*	0.392	0.347
Seasons										
Spring	629	656	89	154	134	48	128	55	10	9
%	39.8	41.5	5.6	9.7	8.5	3	8.1	3.5	0.6	0.6
Summer	1,302	1,335	799	150	155	58	109	43	33	14
%	49.1	50.4	30.1	5.7	5.8	2.2	4.1	1.6	1.2	0.5
Autumn	893	894	310	89	82	268	84	61	26	17
%	46.8	46.9	16.3	4.7	4.3	14.1	4.4	3.2	1.4	0.9
Winter	487	471	55	95	96	46	73	45	9	7
%	41.9	40.5	4.7	8.2	8.3	4	6.3	3.9	0.8	0.6
* P*	<0.01^#^	<0.01^#^	<0.01^#^	<0.01^#^	<0.01^#^	<0.01^#^	<0.01^#^	<0.01^#^	<0.01^#^	0.466

*Der.p, Dermatophagoides pteronyssinus, Der.f, Dermatophagoides farinae.*

Data are presented as number (percentage).

* *P* < 0.05.

# *P* < 0.01.

**Age-related patterns:** Boys outnumbered girls across all three age groups (*P* < 0.01). Significant differences in total sensitization rates were observed among age strata (*P* < 0.01) (Table 1).

**Figure 2:**
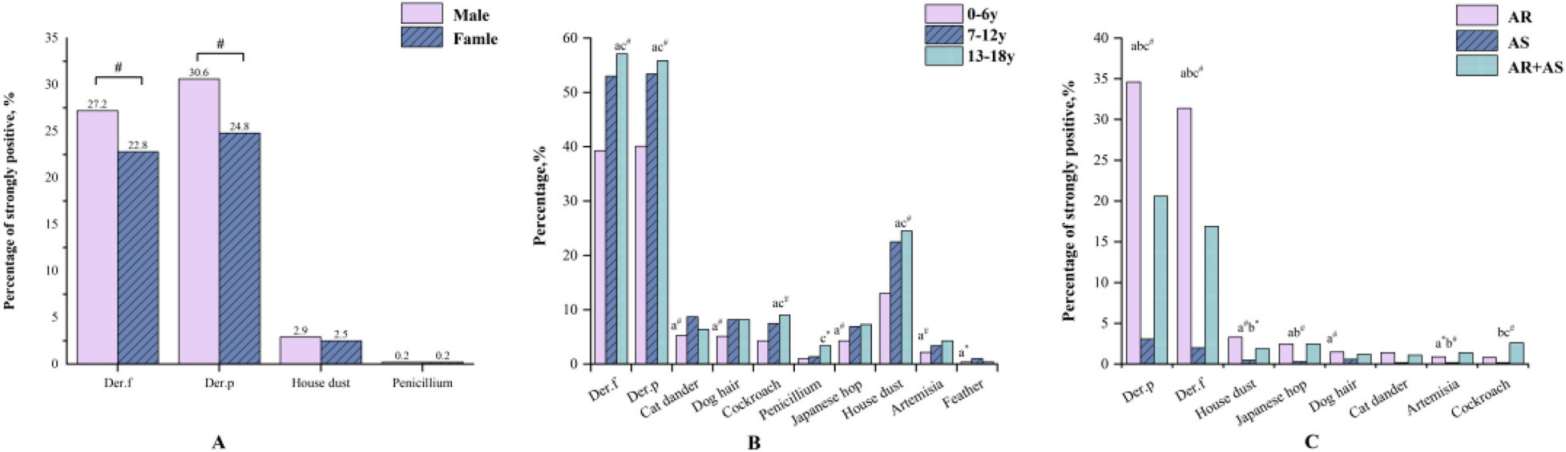
Allergic sensitizations in patients. **(A)** Comparison of strongly positive rates for allergens in different gender groups. **(B)** Comparison of prevalence for allergens in different age groups. **(a)** Compared between 0 and 6 years group and 7–12 years group. **(b)** Compared between 7 and 12 years group and 13–18 years group. **(c)** Compared between 0 and 6 years group and 13–18 years group. **(C)** Comparison of strongly positive rates for allergens in different disease groups. **(a)** Compared between AR group and asthma group. **(b)** Compared between asthma group and AR combined with asthma group. **(c)** Compared between AR group and AR combined with asthma group. *Der.p, Dermatophagoides pteronyssinus, Der.f, Dermatophagoides farinae.* *P < 0.05. ^#^*P* < 0.01.

Sensitization rates to most aeroallergens increased progressively with age, except for cat dander and feather mix. The three allergens with the highest sensitization rates across age groups were *Der.p* (40.1%, 53.4%, and 55.8%), *Der.f* (39.3%, 53.0%, and 57.1%), and house dust (13.0%, 22.5%, and 24.5%). Statistically significant differences in sensitization rates were observed among age groups for these allergens (Figure 2B).

With respect to the number of sensitizing allergens, school-aged children and adolescents had a significantly higher rate of polysensitization than preschool children (Figure 1C).

**Disease-specific profiles:** Male patients predominated in all three clinical disease groups, and significant differences in SPT positive rates were observed among the groups (*P* < 0.01) (Table 1).

Significant differences in sensitization rates to eight aeroallergens were observed in the three disease groups, with the exception of *Penicillium* and feather mix. The three most common aeroallergens in terms of positive rates were *Der.p, Der.f* and house dust (Table 3). Except for dog hair and cat dander, both the AR and AR with AS groups exhibited significantly higher rates of strongly positive reactions to the remaining six allergens compared to the AS group (*P* < 0.05) (Figure 2C).

When comparing the number of allergens to which children were sensitized, significant differences (*P* < 0.05) were observed in both mono- and polysensitization rates among the disease groups. Consistent with the overall sensitization and strongly positive reaction patterns, children with AR or AR with AS comprised a higher proportion of all sensitized cases than those with AS alone (Figure 1D). These findings suggest that sensitization to major aeroallergens, particularly dust mites, may be more strongly associated with upper airway allergic diseases such as allergic rhinitis than with asthma alone.

### 3.3 Geographical and climatic characteristics

A total of 7,301 pediatric patients were classified into three groups based on their residential region: the Sichuan Basin, the southern Sichuan mountainous area, and the northern Sichuan plateau. No significant differences in sex distribution were found. The overall sensitization rate was significantly higher in the Sichuan Basin, which is predominantly plains, compared to the other two regions (Table 1). While sensitization rates to *Penicillium* and feather mix did not differ significantly, statistically significant differences (*P* < 0.05) were observed for the other eight aeroallergens across the three geographical regions. Except for *Japanese hop* and *Artemisia*, which showed the highest sensitization rates in the northwestern Sichuan plateau, others exhibited the highest positive rates in the Sichuan Basin (Table 3).

Based on the visit dates from January 2023 to December 2024, participants were categorized into four seasonal groups: spring (March–May), summer (June–August), autumn (September–November), and winter (December–February). No significant differences in sex distribution were observed across the four seasonal groups, suggesting that the observed seasonal variations in allergen sensitization rates were unlikely to be confounded by differences in sex composition. However, significant seasonal variations were detected in the positive rates for inhalant allergens (Table 1).

Notably, the pattern of seasonal differences in allergen sensitization closely resembled that observed across geographical regions: no significant seasonal variations were found for feather mix, whereas the remaining nine allergens showed statistically significant (*P* < 0.01) differences in sensitization rates across seasons. The positive rates for *Der.f*, *Der.p*, and house dust were highest in summer, followed by autumn, and lowest in spring and winter. In contrast, sensitization rates to dog hair, cat dander, and *Japanese hop* peaked in spring, were moderately high in winter, and lowest in summer and autumn (Table 3).

### 3.4 Multivariable analysis of factors associated with SPT positivity

Multivariable binary logistic regression analysis was conducted to identify factors independently associated with positive SPT results (Table 4). After adjustment for five independent variables, age group, disease type, geographic region, and season remained significantly associated with SPT positivity, whereas sex was not independently associated. Compared with children aged 0–6 years, those aged 7–12 years and 13–18 years showed higher odds of SPT positivity. Disease type and geographic region exhibited strong associations with SPT outcomes, while a borderline seasonal effect was observed for summer testing.

**Table 4:** Multivariable binary logistic regression analysis of factors associated with positive SPT results (*n* = 7,301).

Independent variables	*β*	*P*	*OR*	95% CI
Gender (Male)	−0.012	0.832	0.988	0.885–1.104
Age (0–6)		<0.01		
Age (7–12)	0.734	<0.01	2.083	1.517–2.861
Age (13–18)	0.567	<0.01	1.762	1.577–1.969
Disease (AR)		<0.01		
Disease (AS)	−1.294	<0.01	0.274	0.232–0.324
Disease (AR + AS)	−3.569	<0.01	0.028	0.021–0.037
Geographic regions (Basin)		<0.01		
Geographic regions (Mountain)	0.070	0.792	1.073	0.637–1.807
Geographic regions (Plateau)	2.073	<0.01	7.947	5.252–12.025
Seasons (Spring)		0.007	0.948	0.901–0.998
Seasons (Summer)	−0.174	0.052	0.841	0.706–1.001
Seasons (Autumn)	0.060	0.448	1.062	0.910–1.239
Seasons (Winter)	0.098	0.185	1.103	0.954–1.276

β indicates regression coefficient; *OR* indicates odds ratio; *CI* indicates confidence interval.

Statistical significance was defined as *P* < 0.05.

### 3.5 Association of concomitant atopic diseases with allergen sensitization

Among the 7,301 participants, 1,043 (14.3%) had concomitant atopic diseases, including atopic dermatitis, food allergy, urticaria, and allergic conjunctivitis. No significant differences were observed in age or sex distribution between children with and without other atopic diseases [age: 6 (4–8) vs. 6 (4–8), *P* = 0.505; male: 64.5% vs. 62.3%, *P* = 0.181]. In the unadjusted analyses, children with concomitant atopic diseases showed significantly higher sensitization rates to most aeroallergens (Table 5).

**Table 5:** Association between concomitant atopic diseases and sensitization to aeroallergens.

Allergen	With other atopic diseases (*n* = 1,043)	%	Without other atopic diseases (*n* = 6,258)	%	*P*
*Der.f*	526	50.4	2,785	44.5	<0.001
*Der.p*	562	53.9	2,794	44.6	<0.001
Cat dander	126	12.1	362	5.8	<0.001
Dog hair	116	11.1	351	5.6	<0.001
Cockroach	100	9.6	320	5.1	<0.001
*Penicillium*	21	2.0	57	0.9	0.001
*Japanese hop*	116	11.1	278	4.4	<0.001
House dust	279	26.7	974	15.6	<0.001
*Artemisia*	48	4.6	156	2.5	<0.001
Feather	10	1.0	37	0.6	0.1694

Further multivariable binary logistic regression analysis demonstrated that concomitant atopic diseases remained independently associated with sensitization to multiple major aeroallergens (Table 6). The strongest associations were observed for *Japanese hop* (*OR* = 2.683), cat dander (*OR* = 2.227), *Penicillium* (*OR* = 2.213), and house dust (*OR* = 1.993), after adjustment for age and sex.

**Table 6:** Multivariable binary logistic regression analysis of major aeroallergen sensitization associated with concomitant atopic diseases.

Allergen	Adjusted *OR*	95% *CI*	*P*
*Der.f*	1.264	1.106—1.444	0.001
*Der.p*	1.448	1.267—1.654	<0.001
Cat dander	2.227	1.796—2.762	<0.001
Cockroach	1.965	1.551—2.488	<0.001
*Penicillium*	2.213	1.334—3.670	0.002
*Japanese hop*	2.683	2.135—3.371	<0.001
House dust	1.993	1.706—2.327	<0.001

*OR* indicates odds ratio; *CI* indicates confidence interval.

All models were adjusted for age and sex.

## 4 Discussion

One of the most clinically relevant findings of the present study is the marked predominance of dust mite sensitization, particularly to *Dermatophagoides pteronyssinus* (*Der.p*) and *Dermatophagoides farinae* (*Der.f*), among children with respiratory allergic diseases in Sichuan Province. This pattern is consistent with reports from other Asian countries (19–22) and several regions of China (23–30). Dust mites are thermophilic and hygrophilic organisms that thrive in indoor environments such as bedding, carpets, and upholstered furniture. Sichuan Province (97°21′–108°12′ E, 26°03′–34°19′ N) is characterized by high precipitation, frequent rainfall, limited annual sunshine (only 1,000–1,600 h), and persistently high relative humidity (60%–80%). In particular, the basin-enclosed topography restricts moisture dispersion and creates an ideal microenvironment for dust mite survival and proliferation. This environmental background likely explains the predominance of mite sensitization observed in our cohort. From a clinical perspective, these findings support prioritizing *Der.p* and *Der.f* in routine allergen screening panels and reinforce the importance of early environmental control and mite-specific immunotherapy in this region. In addition, the high prevalence of polysensitization suggests a substantial allergic burden in the pediatric population and may be associated with more complex disease phenotypes (28–31).

Aeroallergens demonstrated distinct spatial and seasonal distributions, further highlighting the influence of climatic and topographical factors on allergen exposure in Sichuan Province. Sensitization to dust mites and pet-related allergens was highest in the Sichuan Basin, where the warm, humid subtropical climate and dense indoor living conditions favor both mite proliferation and indoor allergen accumulation. By contrast, sensitization to *Japanese hop* and *Artemisia* was more prominent in the Western Sichuan Plateau, likely reflecting the regional vegetation profile and ecological environment. *Artemisia* is commonly distributed across alpine meadows and slopes, whereas *Japanese hop* is more likely to grow in moist riparian and valley areas. Together with strong wind exposure that facilitates pollen dispersal, these factors may contribute to the higher pollen-related sensitization rates observed in this region.

Distinct seasonal variation was also observed, with dust mite sensitization peaking in summer, consistent with the hot and humid conditions that facilitate mite growth. In contrast, pet allergen sensitization was relatively higher in spring, which may be partly related to seasonal molting and increased shedding, leading to a greater environmental burden of dander-associated allergens (32). Practically, these findings suggest that allergen screening strategies in Sichuan should be region-specific and season-sensitive. In the basin region, routine screening should prioritize indoor aeroallergens such as *Der.p*, *Der.f*, and house dust, whereas in plateau regions greater attention should be given to outdoor pollen allergens. This regionalized approach may improve screening efficiency and support more targeted prevention and immunotherapy strategies. In addition, the consistently low sensitization rates observed for *Penicillium* and feather mix across demographic and clinical subgroups raise the possibility that their routine inclusion in standard pediatric SPT panels in Sichuan may warrant further evaluation.

Our study further demonstrated that age was an important determinant of allergen sensitization patterns in this cohort, whereas sex was not an independent predictor after adjustment for other demographic and clinical factors. Although boys showed higher sensitization rates in the unadjusted analyses, sex was no longer independently associated with SPT positivity after multivariable adjustment, suggesting that the observed difference may be partly attributable to other demographic and clinical factors.

Age-stratified analysis revealed that sensitization rates to most inhalant allergens increased progressively with age, which is consistent with previous studies and may reflect cumulative environmental exposure together with maturation of the immune system during childhood (33, 34). Preschool children exhibited the lowest overall SPT positivity rates, whereas school-aged children and adolescents showed higher sensitization rates to pet dander and outdoor pollen, together with a greater burden of polysensitization. Several mechanisms may contribute to this age-related trend. Younger children are more likely to be initially sensitized to food allergens because of immature immune and digestive systems, while their exposure to pets and outdoor aeroallergens is often relatively limited (35). As children grow older, broader environmental exposure and repeated allergen contact may contribute to the transition from mono-sensitization to polysensitization (28). In clinical settings, these findings support the need for age-specific allergen screening and prevention strategies in pediatric populations, with particular attention to the early identification of children at risk for progressive sensitization.

Disease-specific differences in allergen sensitization patterns were clinically evident in this study. Children with allergic rhinitis (AR) and those with comorbid AR and asthma showed significantly higher sensitization rates than those with asthma alone, suggesting that inhalant aeroallergens may play a more prominent role in upper airway allergic inflammation. This finding is consistent with the “one airway, one disease” concept and may reflect the direct and repeated exposure of the nasal mucosa to inhaled allergens (36). Notably, polysensitization was more common in children with AR than in those with asthma alone. Previous studies have shown that AR substantially increases the risk of asthma development, particularly in children with multiple allergen sensitizations (37, 38). Therefore, children with AR, especially those with polysensitization, may represent a subgroup at increased risk for asthma progression and warrant earlier intervention and closer longitudinal follow-up. From a therapeutic perspective, these findings also provide important guidance for individualized treatment strategies. In particular, sensitization profile–guided allergen avoidance and allergen-specific immunotherapy may be especially valuable in children with AR and AR combined with asthma, in whom aeroallergen-driven inflammation appears to be more prominent.

Another medically important finding of this study is that children with concomitant atopic diseases exhibited significantly higher sensitization rates to a broad spectrum of aeroallergens. After adjustment for age and sex, concomitant atopic diseases remained independently associated with sensitization to multiple major allergens, particularly *Japanese hop*, cat dander, *Penicillium*, and house dust. These findings support the concept of a shared atopic predisposition and are consistent with the atopic march hypothesis, in which multiple allergic conditions may reflect a heightened Th2-mediated immune response and increased IgE sensitization (39, 40). In clinical settings, children with concomitant atopic diseases may represent a high-risk subgroup for progressive allergic disease and broader sensitization. Therefore, earlier allergen screening, closer longitudinal follow-up, and timely preventive intervention may be particularly important in this population.

In clinical practice, both the skin prick test (SPT) and allergen-specific immunoglobulin E (sIgE) testing are widely used for the assessment of inhalant allergen sensitization. Although both methods are operationally validated for detecting IgE-mediated allergic responses, they differ in their practical applications. SPT is a rapid, cost-effective, and highly sensitive *in vivo* test that reflects immediate hypersensitivity reactions at the skin level and is particularly suitable for routine outpatient screening (41). By contrast, sIgE testing provides a quantitative *in vitro* assessment of circulating allergen-specific IgE antibodies and is less affected by antihistamine use or skin conditions such as eczema. However, it is generally more expensive and may not always correlate directly with clinical symptoms (42, 43). In the present study, SPT was selected as the primary screening method because of its practicality, rapid turnaround, and routine use in pediatric respiratory allergy clinics. Nevertheless, the absence of routine serum sIgE data in this retrospective dataset precluded a direct comparison between these two diagnostic approaches. This limitation should be considered when interpreting the comparative diagnostic implications of the present findings. Future studies integrating both SPT and sIgE measurements may provide a more comprehensive evaluation of sensitization profiles and further improve individualized clinical management.

## 5 Conclusion

In conclusion, this study provides a comprehensive analysis of aeroallergen sensitization patterns among children with respiratory allergic diseases in Sichuan Province. House dust mites, particularly *Dermatophagoides farinae* and *Dermatophagoides pteronyssinus*, were identified as the predominant inhalant allergens, likely influenced by the region's humid basin climate and geographic heterogeneity. Sensitization patterns varied significantly by age, disease phenotype, geographic environment, seasonal distribution, and the presence of concomitant atopic diseases, with both the prevalence of sensitization and the burden of polysensitization increasing with age. Children with allergic rhinitis and those with concomitant atopic diseases exhibited broader and higher rates of sensitization, suggesting a higher-risk subgroup for progressive allergic disease. These findings provide important region-specific evidence for optimized allergen screening, early risk stratification, personalized immunotherapy, and targeted public health interventions in pediatric clinical practice in Sichuan Province.

This study has several limitations. First, its single-center, retrospective design may affect the generalizability of the findings. In addition, serum allergen-specific IgE (sIgE) data were not routinely available for all participants, which precluded a direct comparison between skin prick testing and serological testing methods. However, as a nationally designated children's medical center, the study site provided a large sample covering all pediatric age groups, which strengthens its regional representativeness. Second, the inclusion of only ten allergens may limit the comprehensiveness of the sensitization profile described. Finally, the study did not examine the relationship between sensitization characteristics and clinical disease severity, nor did it perform a detailed correlation between SPT wheal sizes and serum-specific immunoglobulin E (sIgE) levels. Future studies should therefore incorporate broader allergen panels and integrate clinical severity metrics with *in vitro* immunological measures to better characterize the sensitization-disease relationship.

## Data Availability

The raw data supporting the conclusions of this article will be made available by the authors, without undue reservation.
